# Ethnic Disparities of Arrival Following ST Elevation Myocardial Infarction in South Israel

**DOI:** 10.3390/jcm13216516

**Published:** 2024-10-30

**Authors:** Vladimir Zeldetz, Roman Nevzorov, Itai Weissberg, Alan B. Jotkowitz, David Shamia, Tzachi Slutsky, Dan Schwarzfuchs

**Affiliations:** Soroka University Medical Center, Faculty of Health Sciences, Ben Gurion University of the Negev, P.O. Box 151, Beer Shev 84101, Israel; vladimirz@clalit.org.il (V.Z.); alanjot@bgu.ac.il (A.B.J.); dansch@clalit.org.il (D.S.)

**Keywords:** ST elevation myocardial infarction, ethnic disparities, emergency medical service, mode of transportation, primary percutaneous coronary intervention, door-to-balloon time

## Abstract

**Background:** Early activation of the emergency medical service (EMS) is crucial for the care of patients with STEMI. **Methods:** A retrospective cohort study of patients hospitalized with STEMI was conducted. Two groups of patients were compared: Bedouins and Jews. The primary outcome was one-year mortality. The secondary endpoints were 30-day mortality, mode of transportation and door-to-balloon time. **Results:** There were 445 Bedouin patients (BPs) and 1103 Jewish patients (JPs). BPs with STEMI were significantly younger than JPs, had more diabetes and higher rates of smoking. More JPs arrived at the hospital by ambulance compared to BPs (56.9% vs. 31.9%, *p* < 0.001). Direct transportation to the catheterization laboratory was observed in 51.9% of JPs and in 43.6% of BsP, *p* = 0.003. Door-to-balloon time was longer in BPs compared to JPs (median 65 min vs. 62 min, *p* = 0.044). There were no differences in one-year, 30-day and in-hospital mortality between the two groups. After adjustment by propensity score analysis for JPs vs. BPs, there was a protective factor for one-year mortality (hazard ratio (HR), 0.68; 95% CI 0.48–0.97), *p* = 0.034. Thirty-day and one-year mortality in the subgroup of BPs that arrived at the hospital from the outpatient clinic was higher compared to JPs (7.1% vs. 4.4%, *p* = 0.004 and 10.4% vs. 5.6%, *p* < 0.001, relatively) **Conclusions:** Underuse of EMS by BPs with STEMI compared to JPs resulted in a delay in direct transportation to the catheterization laboratory, longer door-to-balloon time and a higher 30-day and one-year mortality in the subgroup of BPs who arrived at the hospital after visiting an outpatient clinic.

## 1. Introduction

The mainstay of STEMI therapy is emergency primary PCI, while thrombolytic therapy remains an initial treatment if the location of the PCI-capable hospital is remote, usually for cases in rural areas. Time to treatment is crucial for the management of patients triaged to the STEMI pathway [1]. It is recommended to perform primary PCI 90 min from arrival to the hospital by both ESC and ACC/AHA/SCAI Guidelines [1,2]. Not infrequently, there is a delay in the pre-hospital setting. A patient delay (the time from symptom onset to the first medical contact) is thought to be multifactorial and associated with socioeconomic factors and sex [3]. A system delay for the patient with suspected STEMI is defined as the time from patient contact with the healthcare system to reperfusion. System delay is a predictor of mortality in STEMI patients handled with primary PCI (PPCI) [4,5,6].

When a presumptive diagnosis of STEMI is made in the pre-hospital setting, early activation of the catheterization laboratory unit diminishes treatment delays and mortality [7,8,9,10,11]. The emergency medical service (EMS) plays a crucial role in the immediate management of patients with suspected STEMI, including determining the primary diagnosis, triage and therapy [4,12].

There are different pathways for arrival at the PCI-capable hospital: from home by private car, from home by ambulance and from home to the outpatient clinic and then to the hospital. After a first medical contact, patients should be referred to the emergency department or go straight to the catheterization lab or to the intensive cardiac care unit.

According to ESC guidelines, when a STEMI presumptive diagnosis is made in the pre-hospital setting and the patient is referred for emergency interventional management, they should bypass the emergency department and proceed directly to the catheterization laboratory. Avoiding the emergency department is associated with a significant reduction in time and may be related to improved survival [13,14,15].

In our area (South of Israel), especially in the Bedouin population, primary care physicians play a central role in the initial treatment of patients with suspected STEMI, probably related to the distance of the PCI-capable hospital and because of less education about ACS. However, in most situations, seeing a general practitioner instead of an immediate call to the ambulance increases the pre-hospital delay.

Earlier studies have recognized ethnic differences in outcomes following acute myocardial infarction. From the National Israeli survey that contained data of 7055 patients (1251, 18% Arabs) hospitalized with ACS. It is known that Israeli Arabs present with ACS at a younger age than non-Arabs and have higher prevalence of smoking and diabetes at presentation [16]. Adjusted 1-year survival was lower among Arab patients despite the similar availability of medical care and in-hospital practices during ACS.

Other studies that evaluated gender and ethnic disparities in outcomes following acute myocardial infarction (AMI) among Bedouins and Jews in Southern Israel included both STEMI and NSTEMI patients, of which only 10% were Bedouins and were performed 14 years ago [17]. Elderly female Bedouins had poor 1-year prognosis following AMI compared with male Bedouins and Jews.

There is a gap in knowledge about patients with STEMI in the growing Bedouin population. In our cohort study, we raised specific questions about the differences in the clinical characteristics, mode of transportation and outcomes focused on patients with STEMI in Jewish and the Bedouin populations.

## 2. Materials and Methods

### 2.1. Patient Population

We performed a retrospective cohort study of patients hospitalized with Acute ST elevation myocardial infarction (STEMI) between 1 January 2016 and 31 December 2022 admitted to the Soroka University Medical Center, a 1200 bed tertiary care teaching medical center that serves as the only regional hospital for southern Israel (Beer-Sheva and vicinity, estimated population of 1,000,000). Bedouins account for 30% of the population.

The goal of the study was a comparison of clinical characteristics, mode of transportation and outcomes of patients with STEMI in Jewish and Bedouin populations. The primary outcome was one-year mortality. The secondary outcomes were in-hospital and 30-day mortality, comparison of clinical characteristics, mode of transportation and door-to-balloon time.

All clinical and demographic characteristics were collected from the electronic hospital medical records.

The exclusion criteria were patients ≤ 18 years old, patients transferred from other hospitals, patients transported by helicopter from rural areas, in-hospital STEMI and non-ST elevation myocardial infarction converted to STEMI.

The study protocol adhered to the Declaration of Helsinki and was approved by the institutional review board (Soroka University Medical Center Institutional Ethics Committee (0298-23-SOR)).

### 2.2. Statistical Analysis

The results are presented as the mean ± SD for continuous variables with normal distribution, as the interquartile range (IQR) for continuous variables with abnormal distribution, and as number and percentage of total patients for categorical data. *t*-test was used for comparison of continuous variables. When the distribution was abnormal, the Mann–Whitney *U* test was applied accordingly. *χ*^2^ test and Fisher’s exact test were used for categorical data. For univariate and multivariate analysis of 1-year survival, Cox proportional hazards regression model was applied. The initial selection of the variables entered in the model was based on univariate analysis significance with inclusion criteria of *p* < 0.1. The results of the Cox proportional hazards model are presented as the hazard ratio (HR) with 95% confidence interval (CI). To adjust for a potential misbalance between Jewish and Bedouin groups in baseline characteristics, a propensity score for nationality was incorporated into the survival model. Moreover, a propensity score matched analysis was performed. Survival curves were calculated by the Kaplan–Meier method and comparison between groups of patients with different risk score was performed by log-rank test. A two-sided *p*-value <0.05 was considered as statistically significant. Statistical analysis was performed with IMB SPSS Statistics software, version 29.0.2.0(20).

## 3. Results

### 3.1. Patient Population

The study cohort included 1548 consecutive patients admitted due to acute ST elevation myocardial infarction, of whom 1103 (71.3%) were Jews and 445 (28.7%) were Bedouins.

**Figure 1:**
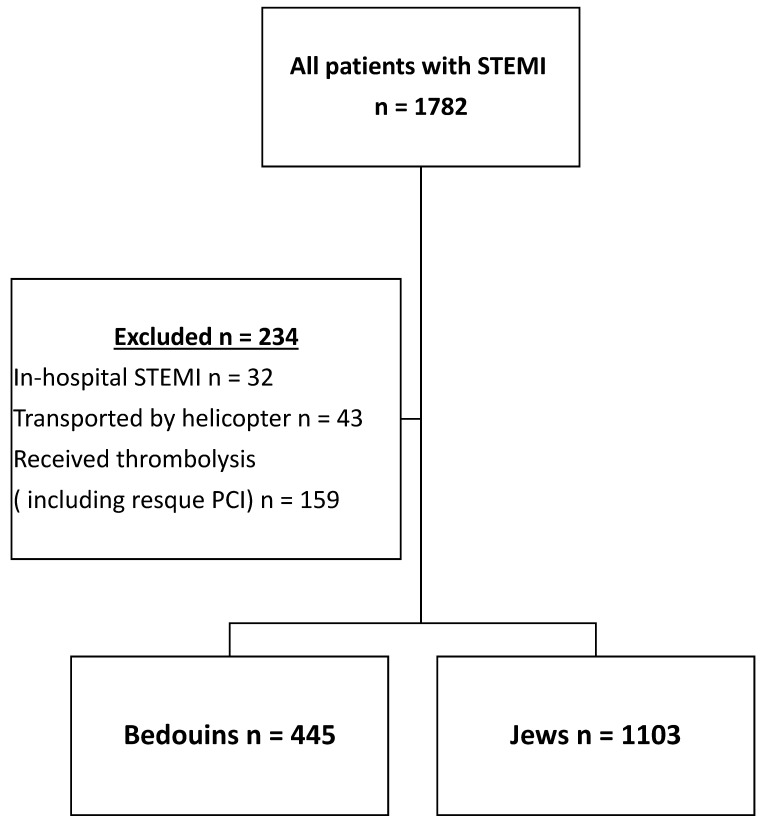
Workflow. Patients with STEMI.

A workflow with included and excluded patients is provided in Figure 1.

Clinical characteristics of the patients (the entire cohort and after propensity score matching) are represented in Table 1. Bedouin patients with STEMI were significantly younger than Jewish patients (56.1 vs. 62.6 years, *p* < 0.001) and included more men compared to the Jewish group (87% vs. 79.9%, *p* < 0.001). Also, there were significantly more patients with diabetes (39.1% vs. 30.5%, *p* < 0.001), history of ischemic heart disease (27.2% vs. 22.2%, *p* = 0.04) and smoking (68.5% vs. 46.9%, *p* < 0.001) in the Bedouin group compared to Jews. Jewish patients had a higher Charlson’s comorbidity index (median score 2 (IQR, 1;3) vs. median sore 2 (IQR 1;4), *p* < 0.001). There was also a trend for slightly higher rates of complete AV block in the Jewish group (3.4% vs. 1.8%, *p* = 0.08) without significant difference in cardiogenic shock and ventricular arrhythmia rate.

Baseline characteristics of Jewish and Bedouin patients were similar after propensity score matching, but the difference in door-to-balloon time and pain-to-balloon time (total ischemic time) disappeared.

### 3.2. Mode of Transportation, Door-to-Balloon Time and Total Ischemic Time

More Jews arrived at the hospital by ambulance compared to Bedouins (56.9% vs. 31.9%, *p* < 0.001). Most of the Bedouin patients visited an outpatient clinic before transportation to the hospital (47.6% vs. 24.9%, *p* < 0.001). A Similar percent of patients (18.1% of Jewish and 20.4% of Bedouin patients, *p* = 0.3) arrived at the hospital by self-transportation.

Direct transportation to the catheterization laboratory or to the intensive cardiac care unit was observed in 51.9% of Jewish patients and in 43.6% of Bedouin patients, *p* = 0.003.

Time from the onset of pain to the first medical contact and time from the onset of pain to arrival at the hospital was not significantly different between the two groups. Time from arrival to hospital to catheterization (in-hospital transportation) was longer in the Bedouin group compared to Jewish group, median 50 min and 47 min, respectively, *p* = 0.019. There was a trend for pain-to-balloon time to be longer in Bedouin patients compared to Jewish patients (median 200 min vs. 190 min, *p* = 0.058). Door-to-balloon time was significantly longer in the Bedouin group compared to the Jewish group (median 65 min vs. 62 min, *p* = 0.044).

Door-to-balloon time was shorter in the subgroup of patients who used EMS, median 55 min, IQR (25:75 Percentiles 40; 75 min) compared to median door to balloon 60 min, IQR (40; 85 min) in the subgroup of those patients who visited the outpatient clinic, *p* = 0.042.

Pain-to-balloon time (total ischemic time) in the subgroup of patients who used EMS was also significantly shorter, median 165 min, IQR (25; 75 Percentiles 130; 237 min) compared to median 245 min, IQR (178; 438 min) in the subgroup of those patients who visited the outpatient clinic, *p* < 0.001.

### 3.3. Outcomes and Predictors for 1-Year Mortality

**Figure 2:**
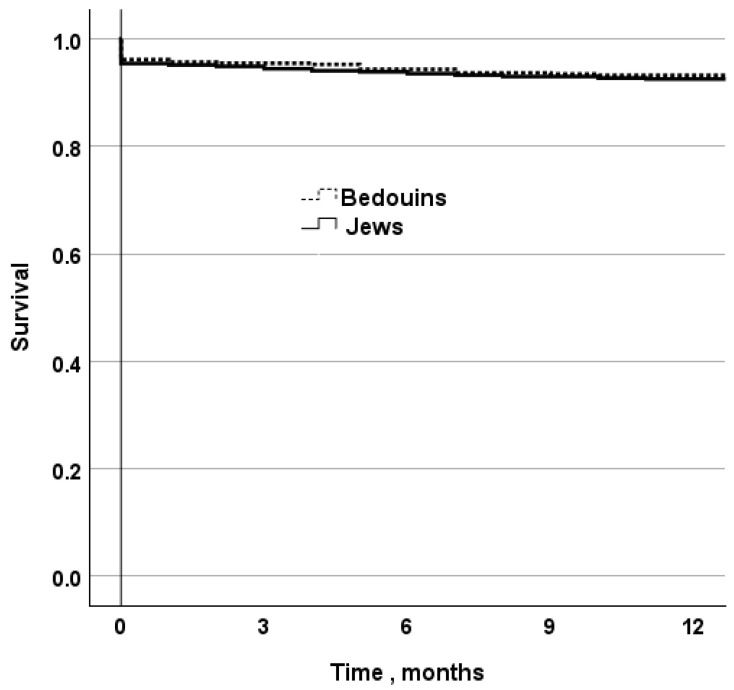
Kaplan–Meier survival plot for one-year survival stratified by nation. Log-rank test *p* = 0.9.

The total 1-year mortality rate of patients with STEMI was the same (10.8%) in both the Jewish and Bedouin groups (Table 2) as shown in Figure 2. There were no significant differences in in-hospital mortality and 30-day mortality between the Bedouin and Jewish groups (6.1% vs. 6.2, *p* = 0.9 and 7% vs. 6.4%, *p* = 0.3, relatively). Thirty-day mortality of Bedouin patients that arrived at the hospital from the outpatient clinic was significantly higher compared to Jewish patients (7.1% vs. 4.4%, *p* = 0.004) and 1-year mortality rate in the same subgroups remained the significant difference (10.4% vs. 5.6%, *p* < 0.001). There was a trend for a higher 30-day mortality and 1-year mortality in Jewish patients arriving at the hospital by self-transportation compared to Bedouin patients (5% vs. 2.2%, *p*-value = 0.3 and 9% vs. 2.2%, *p* = 0.06) that did not reach statistical significance possibly due to small numbers.

However, after applying propensity score analysis to adjust for a potential misbalance between the Jew and Bedouin groups in baseline characteristics and the possible confounders, we found the Jew group vs. Bedouin ethnical group was a protective factor for one-year mortality (hazard ratio (HR), 0.68; 95% CI 0.48–0.97), *p* = 0.034, and a trend for being protective with borderline significance in 30-day mortality (HR, 0.65; 95% CI 0.42–1.004, *p* = 0.052).

Cardiogenic shock, higher Charlson comorbidity index and severe left ventricular dysfunction were found as independent predictors for 1-year mortality (Table 3).

## 4. Discussion

Our study compared the clinical characteristics, mode of transportation and outcomes of patients with STEMI in Jewish and Bedouin populations.

First, we found that Bedouin patients with STEMI were significantly younger and more likely to smoke and to have diabetes than Jewish patients. Baseline characteristics of Jewish and Bedouin patients were similar after propensity score matching. The high percentage of diabetes mellitus in the Bedouin group may represent a transition of this population from a traditional nomadic lifestyle to a Western sedentary one, characterized by changes in dietary habits and a reduction in physical activity [18].

Second, in contrast to Jewish patients with STEMI, Bedouin patients preferred to visit an outpatient clinic first and then were referred to the hospital, which contributed to the delay in primary invasive therapy and reperfusion. A probable explanation could be cultural factors and the relatively remote distance from the PCI-capable hospital.

Nevertheless, a time to the first medical contact and the symptom-onset to hospital-arrival time were similar in both groups but the door-to-balloon time was significantly shorter in Jewish patients that used ambulances more frequently to get to the hospital. We also demonstrated the underuse of ambulances in the Bedouin population. This fact led to a delay in receiving rapid initial therapy and more critically prevented the bypassing of the emergency department. As a result, 51.9% of Jewish patients vs. only 43.6% Bedouin patients (*p* = 0.003) were transferred directly to the catheterization lab.

There were comparable mortality rates in Jewish and Bedouin patients; while Bedouin patients were significantly younger, they had more comorbidities such as diabetes mellitus, smoking and previously known coronary disease.

However, after applying propensity score analysis to adjust for a potential misbalance between Jews and Bedouin groups in baseline characteristics and the possible confounders, we found the Jewish group vs. Bedouin ethnical group was a protective factor for one-year mortality (hazard ratio (HR), 0.68; 95% CI 0.48–0.97), *p* = 0.034, and a trend for being protective with borderline significance in 30-day mortality (HR, 0.65; 95% CI 0.42–1.004, *p* = 0.052).

In the analysis of mortality rates by mode of transportation, we discovered that the 30-day mortality of Bedouin patients that arrived at the hospital after visiting an outpatient clinic was significantly higher compared to Jewish patients (7.1% vs. 4.4%, *p* = 0.004) and 1-year mortality rate in the same subgroups remained significantly different (10.4% vs. 5.6%, *p* < 0.001). In the subgroup of patients that arrived at the hospital by self-transportation, there was a trend for higher 30-day and 1-year mortality than in the Jewish group. Surprisingly, almost 20% of both Jewish and Bedouin patients with STEMI reached the hospital by self-transportation.

Both door-to-balloon time and pain-to-balloon time (total ischemic time) were significantly shorter in the subgroups of patients who used EMS directly compared to the subgroup of patients who visited the outpatient clinic, reflecting the delay not only in the pre-hospital setting but also a contribution to the intra-hospital delay in cases when EMS was not activated.

A few previous studies evaluated the differences among patients with STEMI who were transported to the hospital by emergency medical services (EMSs) or by private vehicles or were transferred from other medical facilities.

Callachan EL et al., in a study of 455 STEMI patients in the United Arab Emirates, found that EMS transportation was associated with a shorter time to treatment including symptom-onset-to-balloon time and door-to-balloon time [19].

Najafi H et al. performed a population-based comparison of the outcomes of EMS vs. non-EMS transport of 2244 patients with STEMI in Southern Iran [20]. The death rate in patients who used EMS transport was lower than those who used non-EMS transport.

Mathews et al. analyzed data derived from a large American STEMI patient cohort of 37,634 patients treated in 372 US hospitals participating in the National Cardiovascular Data Registry Acute Coronary Treatment and Intervention Outcomes Network Registry [21]. The investigators found that EMS transport was used in only 60% of STEMI patients. EMS-transported patients compared with self-transported patients had significantly shorter delays in both symptom-onset-to-arrival time and door-to- reperfusion time.

Newport R et al. in a recent systematic review assessed the ethnic differences of the care pathway following an out of hospital cardiac event [22]. The authors identified 3552 related articles and attempted to perform a meta-analysis of 40 eligible for the review articles but discovered significant heterogeneity across the studies so that the meta-analysis was not performed. It was concluded that the extant reasons for differences in cardiac care pathways are considerable.

It should be mentioned that in our study we had a unique opportunity to compare Bedouin and Jewish populations with STEMI. Bedouin patients with STEMI were significantly younger so that there should be more efforts for cardiovascular prevention in this population. Patient education may play an important role in decreasing patient delays and using the ambulance after the onset of chest pain suspicious for ACS.

Probably, our study does not contain any novelty in terms of geographical differences in STEMI treatment. However, it may underline the importance of a more aggressive approach and focused diagnostic workup even in unfavorable settings.

### Limitations

Our study has several limitations, involving a single center and being retrospective in nature with a relatively small sample size. Some findings may represent a local phenomenon. Also, we did not analyze initial therapy received after first medical contact and thereafter. In addition, we did not investigate catheterization findings with subsequent revascularization procedures, vascular access and periprocedural complications, and compliance with medical therapy including dual antiplatelet therapy. Some patients might need both anticoagulation and antithrombotic therapy; thus, the increasing risk of bleeding complications might be partially responsible for outcomes. We did not analyze the type of treatment that was given according to the ESC guidelines. Finally, the COVID-19 pandemic could lead to lower access to healthcare and cause a treatment delay remote from the center area.

## 5. Conclusions

Underuse of EMS by Bedouin patients with STEMI compared to Jewish patients resulted in a delay in direct transportation to the catheterization laboratory, longer door-to-balloon time and a significantly higher 30-day and one-year mortality in the subgroup of Bedouin patients who arrived at the hospital after visiting an outpatient clinic. After applying propensity score analysis to adjust for a potential misbalance between Jews and Bedouin groups in baseline characteristics, the Jewish group vs. the Bedouin group was a protective factor for one-year mortality.

Bedouin patients with STEMI were significantly younger so that intensive efforts in patient education and cardiovascular prevention are crucially important in this high-risk population.

## Figures and Tables

**Table 1:** Clinical characteristics.

Characteristics	Jews *n* = 1103	Bedouins *n*= 445	*p*-Value	Jews (After Propensity Score Matching) *n* = 445	Bedouins *n* = 445	*p* = Value
Age, years, mean ± SD	62.6 ± 12.6	56.1 ± 12.6	<0.001	61± 13.3	56.1 ± 12.6	<0.001
Male, *n* (%)	881 (79.9)	387 (87)	<0.001	362 (81.3)	387 (87)	0.02
Ischemic heart disease, *n* (%)	245 (22.2)	121 (27.2)	0.04	96 (21.6)	121 (27.2)	0.06
Hypertension, *n* (%)	548 (49.7)	198 (44.5)	0.06	221 (49.7)	198 (44.5)	0.1
Diabetes mellitus, *n* (%)	336 (30.5)	174 (39.1)	0.001	125 (28.1)	174 (39.1)	<0.001
Smoking, *n* (%)	517 (46.9)	305 (68.5)	<0.001	200 (44.9)	305 (68.5)	<0.001
Dyslipidemia, *n* (%)	752 (68.2)	305 (68.5)	0.9	291 (65.4)	305 (68.5)	0.3
Charlson’s score, median, IQR	2 (1; 4)	2 (1; 3)	<0.001	2 (1; 4)	2 (1; 3)	0.02
Direct Arrival to Catheterization laboratory or ICCU, *n* (%)	572 (51.9)	194 (43.6)	0.003	251 (56.4)	194 (43.6)	0.1
Pathways of arrival to the hospital						
Self-transportation, *n* (%)	200 (18.1)	91 (20.4)	0.3	91 (20.4)	91 (20.4)	1
Arrival by ambulance, *n* (%)	628 (56.9)	142 (31.9)	<0.001	219 (49.2)	142 (31.9)	<0.001
Arrival from outpatient clinic, *n* (%)	275 (24.9)	212 (47.6)	<0.001	135 (30.3)	212 (47.6)	<0.001
Time to first medical contact, min, median (IQR)	80 (40; 180)	90 (45; 195)	0.17	85 (40; 190)	90 (45; 195)	0.3
Symptom-onset to hospital-arrival time, min, median (IQR)	120 (75; 227)	130 (80; 240)	0.16	122 (70; 225)	130 (80; 240)	0.2
Time from arrival to hospital to catheterization, min, median (IQR)	47 (30; 70)	50 (30; 76)	0.019	47 (47; 69)	50 (30; 76)	0.048
Door-to-balloon time, min, median (IQR)	62 (44; 85)	65 (45; 89)	0.044	62 (45; 85)	65 (45; 89)	0.1
Pain-to-balloon time, min, median (IQR)	190 (140; 302)	200 (147; 305)	0.058	195 (137; 302)	200 (147; 305)	0.7
Significant left ventricular dysfunction, *n* (%)	601 (54.5)	256 (57.5)	0.3	254 (57.1)	256 (57.5)	0.9
Sustained ventricular tachycardia or ventricular fibrillation, *n* (%)	94 (8.5)	50 (11.2)	0.1	38 (8.5)	50 (11.2)	0.1
Complete atrioventricular block, *n* (%)	37 (3.4)	8 (1.8)	0.084	8 (1.8)	8 (1.8)	1
Cardiogenic Shock, *n* (%)	61 (5.5)	21 (4.7)	0.5	22 (4.9)	21 (4.7)	0.8

**Table 2:** Outcomes.

Outcomes	Jews *n* = 1103	Bedouins *n*= 445	*p*-Value
30-day mortality, *n* (%)	71 (6.4)	31 (7.0)	0.3
Self-transportation, *n* (%)	10 (5)	2 (2.2)	0.3
Arrival by ambulance, *n* (%)	49 (7.8)	14 (9.9)	0.3
Arrival from outpatient clinic, *n* (%)	12 (4.4)	15 (7.1)	0.004
In- hospital ortality, *n* (%)	68 (6.2)	27 (6.1)	0.9
Self-transportation, *n* (%)	10 (5)	2 (2.2)	0.3
Arrival by ambulance, *n* (%)	47 (7.5)	13 (9.2)	0.5
Arrival from outpatient clinic, *n* (%)	11 (4)	12 (5.7)	0.4
One-year mortality, *n* (%)	119 (10.8)	47 (10.8)	0.96
Self-transportation, *n* (%)	18 (9)	2 (2.2)	0.06
Arrival by ambulance, *n* (%)	85 (13.5)	20 (14.1)	0.8
Arrival from outpatient clinic, *n* (%)	16 (5.8)	22 (10.4)	<0.001

**Table 3:** Multivariate analysis of factors associated with one-year mortality.

Factor	Hazard Ratio	95% CI	*p*-Value
Cardiogenic Shock	7.8	5.6–11.1	<0.001
Charlson comorbidity index, increment for each point	1.5	1.4–1.7	<0.001
Significant left ventricular dysfunction vs. mild dysfunction or preserved systolic function	3.2	2.1–4.9	<0.001

## Data Availability

The datasets presented in this article are not readily available due to privacy and ethical restrictions.

## References

[1] Byrne R.A., Rossello X., Coughlan J.J., Barbato E., Berry C., Chieffo A., Claeys M.J., Dan G.A., Dweck M.R., Galbraith M. (2023). 2023 ESC Guidelines for the management of acute coronary syndromes. Eur. Heart J..

[2] Lawton J.S., Tamis-Holland J.E., Bangalore S., Bates E.R., Beckie T.M., Bischoff J.M., Bittl J.A., Cohen M.G., DiMaio J.M., Don C.W. (2022). 2021 ACC/AHA/SCAI Guideline for Coronary Artery Revascularization: A Report of the American College of Cardiology/American Heart Association Joint Committee on Clinical Practice Guidelines. Circulation.

[3] Jackson A.M., Zhang R., Findlay I., Robertson K., Lindsay M., Morris T., Forbes B., Papworth R., McConnachie A., Mangion K. (2020). Healthcare disparities for women hospitalized with myocardial infarction and angina. Eur. Heart J. Qual. Care Clin. Outcomes.

[4] Terkelsen C.J., Sørensen J.T., Maeng M., Jensen L.O., Tilsted H.H., Trautner S., Vach W., Johnsen S.P., Thuesen L., Lassen J.F. (2010). System delay and mortality among patients with STEMI treated with primary percutaneous coronary intervention. JAMA.

[5] Jortveit J., Pripp A.H., Halvorsen S. (2022). Outcomes after delayed primary percutaneous coronary intervention vs. pharmaco-invasive strategy in ST-segment elevation myocardial infarction in Norway. Eur. Heart J. Cardiovasc. Pharmacother..

[6] Larsen A.I., Løland K.H., Hovland S., Bleie Ø., Eek C., Fossum E., Trovik T., Juliebø V., Hegbom K., Moer R. (2022). Guideline-recommended time less than 90 minutes from ECG to primary percutaneous coronary intervention for ST-segment-elevation myocardial infarction is associated with major survival benefits, especially in octogenarians: A contemporary report in 11,226 patients from NORIC. J. Am. Heart Assoc..

[7] Kontos M.C., Gunderson M.R., Zegre-Hemsey J.K., Lange D.C., French W.J., Henry T.D., McCarthy J.J., Corbett C., Jacobs A.K., Jollis J.G. (2020). Prehospital activation of hospital resources (PreAct) ST-segment-elevation myocardial infarction (STEMI): A standardized approach to prehospital activation and direct to the catheterization laboratory for STEMI recommendations from the American Heart Association’s mission: Lifeline program. J. Am. Heart Assoc..

[8] Squire B.T., Tamayo-Sarver J.H., Rashi P., Koenig W., Niemann J.T. (2014). Effect of prehospital cardiac catheterization lab activation on door-to-balloon time, mortality, and false-positive activation. Prehosp. Emerg. Care.

[9] Fordyce C.B., Al-Khalidi H.R., Jollis J.G., Roettig M.L., Gu J., Bagai A., Berger P.B., Corbett C.C., Dauerman H.L., Fox K. (2017). Association of rapid care process implementation on reperfusion times across multiple ST-segment-elevation myocardial infarction networks. Circ. Cardiovasc. Interv..

[10] Shavadia J.S., Roe M.T., Chen A.Y., Lucas J., Fanaroff A.C., Kochar A., Berger P.B., Corbett C.C., Dauerman H.L., Fox K. (2018). Association between cardiac catheterization laboratory pre-activation and reperfusion timing metrics and outcomes in patients with ST-segment elevation myocardial infarction undergoing primary percutaneous coronary intervention: A report from the ACTION registry. JACC Cardiovasc. Interv..

[11] Stowens J.C., Sonnad S.S., Rosenbaum R.A. (2015). Using EMS dispatch to trigger STEMI alerts decreases door-to-balloon times. West J. Emerg. Med..

[12] Huber K., De Caterina R., Kristensen S.D., Verheugt F.W.A., Montalescot G., Maestro L.B., Werf F.V.D. (2005). Pre-hospital reperfusion therapy: A strategy to improve therapeutic outcome in patients with ST-elevation myocardial infarction. Eur. Heart J..

[13] Meisel S.R., Kleiner-Shochat M., Abu-Fanne R., Frimerman A., Danon A., Minha S., Levi Y., Blatt A., Mohsen J., Shotan A. (2021). Direct admission of patients with ST-segment-elevation myocardial infarction to the catheterization laboratory shortens pain-to-balloon and door-to-balloon time intervals but only the pain-to-balloon interval impacts short- and long-term mortality. J. Am. Heart Assoc..

[14] Bagai A., Jollis J.G., Dauerman H.L., Peng S.A., Rokos I.C., Bates E.R., Bates E.R., French W.J., Granger C.B., Roe M.T. (2013). Emergency department bypass for ST-segment-elevation myocardial infarction patients identified with a prehospital electrocardiogram: A report from the American Heart Association Mission: Lifeline program. Circulation.

[15] Scholz K.H., Friede T., Meyer T., Jacobshagen C., Lengenfelder B., Jung J., Fleischmann C., Moehlis H., Olbrich H.G., Ott R. (2020). Prognostic significance of emergency department bypass in stable and unstable patients with ST-segment elevation myocardial infarction. Eur. Heart J. Acute Cardiovasc. Care.

[16] Karkabi B., Zafrir B., Jaffe R., Shiran A., Jubran A., Adawi S., Ben-Dov N., Iakobishvili Z., Beigel R., Cohen M. (2020). Ethnic Differences Among Acute Coronary Syndrome Patients in Israel. Cardiovasc. Revasc. Med..

[17] Plakht Y., Gilutz H., Shiyovich A., Zahger D., Weitzman S. (2011). Gender and ethnic disparities in outcome following acute myocardial infarction among Bedouins and Jews in southern Israel. Eur. J. Public Health.

[18] Cohen A.D., Gefen K., Ozer A., Bagola N., Milrad V., Cohen L., Abu-Hammad T., Abu-Rabia Y., Hazanov I., Vardy D.A. (2005). Diabetes control in the Bedouin population in southern Israel. Med. Sci. Monit..

[19] Callachan E.L., Alsheikh-Ali A.A., Nair S.C., Bruijns S., Wallis L.A. (2017). Outcomes by Mode of Transport of ST Elevation MI Patients in the United Arab Emirates. West. J. Emerg. Med..

[20] Najafi H., Bahramali E., Bijani M., Dehghan A., Amirkhani M., Balaghi Inaloo M. (2022). Comparison of the outcomes of EMS vs. Non-EMS transport of patients with ST-segment elevation myocardial infarction (STEMI) in Southern Iran: A population-based study. BMC Emerg. Med..

[21] Mathews R., Peterson E.D., Li S., Roe M.T., Glickman S.W., Wiviott S.D., Saucedo J.F., Antman E.M., Jacobs A.K., Wang T.Y. (2011). Use of emergency medical service transport among patients with ST-segment-elevation myocardial infarction: Findings from the National Cardiovascular Data Registry Acute Coronary Treatment Intervention Outcomes Network Registry-Get with the Guidelines. Circulation.

[22] Newport R., Grey C., Dicker B., Ameratunga S., Harwood M. (2023). Ethnic differences of the care pathway following an out-of-hospital cardiac event: A systematic review. Resuscitation.

